# Functional evaluation and testing of a newly developed Teleost’s Fish Otolith derived biocomposite coating for healthcare

**DOI:** 10.1038/s41598-019-57128-w

**Published:** 2020-01-14

**Authors:** Nerly D. Montañez, Heider Carreño, Patricia Escobar, Hugo A. Estupiñán, Darío Y. Peña, Saurav Goel, Jose L. Endrino

**Affiliations:** 1 Corrosion Research Group GIC, Universidad Industrial de Santander, Piedecuesta, 681011 Colombia; 2 Center for Research in Tropical Diseases CINTROP, Universidad Industrial de Santander, Piedecuesta, 681011 Colombia; 3 Biomaterials Laboratory, Universidad Nacional de Colombia, Medellín, 050034 Colombia; 4 School of Aerospace, Transport and Manufacturing, Cranfield University, Bedford, MK43 0AL UK; 5 Basque Center for Materials, Applications & Nanostructures, UPV/EHU Science Park, Barrio Sarriena s/n, 48940 Leioa, Spain; 6 IKERBASQUE, Basque Foundation for Science, Maria Diaz de Haro 3, 48013 Bilbao, Spain; 7 School of Engineering, London South Bank University, 103 Borough Road, London, SE1 0AA UK

**Keywords:** Biomaterials, Materials for devices

## Abstract

Polymers such as polycaprolactone (PCL) possess biodegradability, biocompatibility and affinity with other organic media that makes them suitable for biomedical applications. In this work, a novel biocomposite coating was synthesised by mixing PCL with layers of calcium phosphate (hydroxyapatite, brushite and monetite) from a biomineral called otolith extracted from Teleost fish (Plagioscion Squamosissimus) and multiwalled carbon nanotubes in different concentrations (0.5, 1.0 and 1.5 g/L). The biocomposite coating was deposited on an osteosynthesis material Ti6Al4V by spin coating and various tests such as Fourier transformation infrared spectroscopy (FTIR), Raman spectroscopy, scanning electron microscopy (SEM), transmission electron microscopy (TEM), scratch tests, MTT reduction cytotoxicity, HOS cell bioactivity (human osteosarcoma) by alkaline phosphatase (ALP) and fluorescence microscopy were performed to comprehensively evaluate the newly developed biocoating. It was found that an increase in the concentration of carbon nanotube induced microstructural phase changes of calcium phosphate (CP) leading to the formation of brushite, monetite and hydroxyapatite. While we discovered that an increase in the concentration of carbon nanotube generally improves the adhesion of the coating with the substrate, a certain threshold exists such that the best deposition surfaces were obtained as PCL/CP/CNT 0.0 g/L and PCL/CP/CNT 0.5 g/L.

## Introduction

Metallic biomaterials used in implantology are usually inert, since there is no ion active exchange. This property allows the cells to recognize the microenvironment, proliferate and differentiate in a better way. For this reason, biocompatible materials in coatings such as calcium ceramics with a chemical nature like bone, or natural or synthetic polymers allows easy incorporation of bioactive materials or active ingredients making the recovery of hard tissue faster.

**Figure 1:**
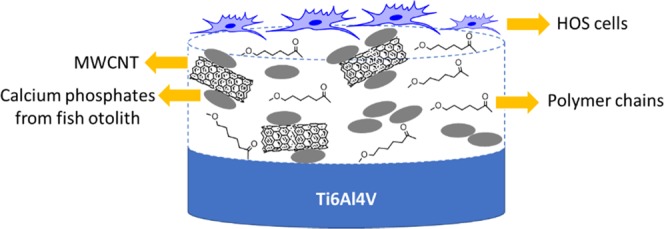
Multiwalled carbon nanotubes functionalized with CP in a PCL polymer matrix coating.

One of the widely used polymers in tissue engineering is polycaprolactone (PCL - poly ε-caprolactone). It possesses semi-crystalline state, hydrophobic character, good solubility, low melting point, adequate degradation rate and excellent compatibility^1–3^, making it a good candidate for use as a biocoating material. One drawback as with many other polymers that the PCL has is its low mechanical strength which is proposed to be modified in this paper via addition of functionalised multiwalled carbon nanotubes (MWCNT) (Fig. 1). Recently, it has been shown that PCL and carbon nanotubes can forms an interface that improves certain properties in the composite^4^. Besides, carbon nanotubes can be functionalized, allowing other molecules to be adhered to the walls and ends, improving the properties of the materials. This strategy has been widely used to incorporate calcium phosphates (CP) like hydroxyapatite^5–7^, CaCO_3_^2^ or polymers for applications such as cancer treatment, drug transport, biotechnological applications and energy^8–10^. As Surmenev *et al*. indicate, much of the *in vitro* results of inorganic Ca-P coatings show that they promote bone regeneration. However, other studies such as that of Mokabber *et al*. show that, depending on the morphology of the coating, the cell adhesion process varies, since cells are not viable on rough surfaces due to physical damage caused by sharp ribbons and needles in electrodeposited coatings of Ca-P. In addition, the *in vitro* results about the effectiveness of Ca-P coatings are contradictory, because the mechanism of osteogenesis is still unclear^11,12^. Therefore, the use of a composite material using polymers may favour the biocompatibility response.

There are scarce studies focussing on functionalised carbon nanotubes with calcium phosphates in a polymer matrix that are reviewed here. Grandfield *et al*.^13^ incorporated in a matrix of chitosan, hydroxyapatite nanoparticles and carbon nanotubes, and then electrodeposited this material on Nitinol, concluding that these layers protect Nitinol from corrosion, however, functionalization of carbon nanotubes was not studied. Nawrotek *et al*.^14^ electrodeposited chitosan-carbon nanotubes with calcium ions and reported that CNT improves the mechanical properties and that the concentrations used of 10 mg and 100 mg did not affect the cytotoxicity of the composite. Cai *et al*.^15^ functionalized carbon nanotubes with calcium phosphate, verifying the adherence of the phosphates to the CNT. This material was added to a PLLA (polylactic acid) matrix to obtain PLLA-HA-CNT scaffolds, where the mechanical properties of the composite material were found to be improved.

The objective of this study was to evaluate the performance improvement in the strength of PCL polymer coated with functionalised multiwall carbon nanotubes. As for the functionalisation, a specialised calcium based ceramic material was obtained from Otolith extracted from Teleost Fish (Plagioscion Squamosissimus). The characterization and synthesis of calcium phosphates from this natural material was studied in a previous work^14^. It comes from an organic matrix that provides a favour for the nucleation of aragonite crystals. This material was incorporated into a PCL polymer matrix to be used as a coating on Ti6Al4V substrate. The physicochemical properties were evaluated, as well as the scratch test that allowed to verify the adherence of the coating to the substrate. A cell culture of HOS cells (human osteosarcoma) was carried out to verify the cytotoxicity, production of alkaline phosphatase and cellular adhesion of the PCL/CP/CNT composite in different concentrations of nanotubes. This study allowed observing the behaviour of the material from various points of view to propose it or not as a potential material in orthopaedic applications.

The biomineral Otolith was obtained from the fish merchants since it is not consumed, and in other cases it is discarded by the consumer, so this investigation had no contact with the living or dead animal species, nor caused its unnecessary death. Due to the use of the Otolith, this research requested endorsement from the Committee of Ethics in Scientific Research of the Universidad Industrial de Santander, which was granted by act number 25 of 16th December 2016, following the institutional guideline Guide Ethical Considerations Animals GIN.09.

## Material and Methods

### Functionalization of multi-walled carbon nanotubes/calcium phosphate from otolith of a fish

Multiwalled carbon nanotube (MWCNT) with diameter 6 to 13 nm and length 2.5 to 20 μm procured from Sigma Aldrich (CAS number 308068-56-6) and polycaprolactone (Sigma Aldrich CAS number 24980-41-4 Mn 80000) were used to functionalise. To improve the solubility of the nanotubes in organic solutions, they were exposed to an acid - base treatment with HNO_3_, NaOH and HCl for elimination of impurities^16^. Functionalization of carbon nanotubes with calcium phosphates was carried out by aqueous precipitation process of calcium phosphates. The MWCNT in three concentrations were added in a solution of calcium hydroxide (Ca(OH)_2_) 1 M, followed by dispersion in ultrasound for 20 minutes at an amplitude of 45 kHz and pulses 2:1 using the Sonics Vibra Cell VCX500 equipment. A solution of 0.6 M phosphoric acid was mixed to the previous solution u at a rate of 5 ml/min and a temperature of 90 °C for one hour and 25 °C for an additional hour with stirring of 400 rpm until reaching a pH value of 4.0. The resulting solution precipitated for 48 hours, filtered, washed and dried at 120 °C for 24 hours. The final concentration of carbon nanotubes was 0.5, 1.0 and 1.5 g/L. Ca(OH)_2_ was obtained from natural aragonite (CaCO_3_) at 82.5 wt% (fish otolith) according to the procedure described by Montañez *et al*.^17^

### Characterization of MWCNT/CP powders

The chemical characterization of functionalization of MWCNT with calcium phosphates (CP) was performed by Raman spectroscopy (Labram HR Evolution HORIBA Scientific) at room temperature using a 532 nm laser using a power of 50 mW, acquisition time of 7 s and accumulation time of 5 s. Fourier transform infrared spectroscopy (FTIR) on a Nicolet iS50 spectrometer with the total attenuated reflection technique ATR in a range of 4000 to 400 cm^−1^, with 32 scans at a resolution of 4 cm^−1^ was also carried out. The morphology was observed in a scanning electron microscope (SEM) - FEI Quanta 650, secondary electrons, with a working distance of 8.5 mm and an acceleration voltage of 15 kV and transmission electron microscopy (TEM) was performed in a Tecnai F20 Super Twin TMP-FEI device performing the dispersion of CP/MWCNT in ethanol, immersing the solution in an ultrasound bath for 5 minutes. The software used to create the legends in the micrographs was ImageJ version number 1.52a of National Institutes of Health, USA (https://imagej.nih.gov/ij/). ImageJ is in the public domain.

### Preparing polycaprolactone coatings reinforced with CP/MWCNT from fish otoliths by spin coating

Polycaprolactone (PCL) and acetic acid were used as solvents which were prepared at 2.5% w/V by magnetic stirring for 4 hours. An amount of 4 g/L of CP/MWCNT was added to the previous solution to obtain solutions of polycaprolactone/CP/MWCNT with different concentrations of carbon nanotubes. An ultrasound probe was used at an amplitude of 45% for 5 minutes to disperse CP/MWCNT in the PCL solution. The coatings were obtained by the spin coating technique, using a Ti6Al4V alloy of 13 mm diameter and 3 mm thickness as a substrate with a surface prepared with silicon carbide paper number 180, 240, 360, 400 and 600 and chemically treated in accordance with ASTM E407-07 (2015) for 20 seconds. The conditions for spin coating were 3000 rpm for the first layer and 4000 rpm for the following layers. Five layers were deposited, using 30 μl each, 60 seconds and 10 minutes of drying at 50 °C between layer and layer. As a final step, the Ti6Al4V samples were cleaned in ethanol and deionized water using an ultrasonic bath for 10 minutes. The characterization of coatings was performed by SEM, FTIR and Raman spectroscopy at a time of acquisition of 10 s and accumulation time of 6 s.

### Scratch tests

Scratch tests were carried out on TEER scratch tester ST3001 with a diamond tip (Rockwell C, 200 µm radius tip), under continuous progressive load (4 to 50 N) at a loading rate of 46 N/min and a sliding speed of 5 mm/min for an overall scratch length of 5 mm. The friction coefficient was calculated with obtaining friction force (N) and normal force (N) acting on the scratch tip. The friction force and the first derivate of friction force versus normal load allowed to determine the critical load (Lc_3_) as the normal load at which the adhesive failure of the coating occurred. Micrographs of the total scratch length and the failure point were captures via a Nikon stereomicroscope image acquisition system and microscope Leica DM 2700 M respectively, both with software LAS (Leica Application Suite V 4.8). All tests were performed in triplicate.

### Cell culture

The human osteosarcoma cell line (HOS, ATCC CRL-1543) was maintained by serial passages in RPMI-1640 medium supplemented with 10% of heat inactivated foetal bovine serum (_hi_SFB) and penicillin/streptomycin at 5% CO_2_ and 95% humidity at 37 °C. For subculture, cells were detached from bottle surface using a solution of trypsin (0.25% w/v) - EDTA (0.53 mM) for 10 minutes at 37 °C, washed with culture medium, centrifuged and resuspended with fresh medium.

### Toxicity test

Each of the coating samples were left in a 24 well plate flat bottom plate containing 1 mL of RPMI-1640–10% _hi_SFB at 37 °C. After 30 days, supernatants were recollected and stored at −20 °C before use. HOS cells (5 × 10^4^ cells/mL), were incubated with serial dilutions of each supernatants or medium alone for 72 h. Cell toxicities were assessed using a colorimetric MTT test and percentage of cell viability was calculated by the formula Cell viability (%) = Average OD in study group/average OD in control group × 100%, where OD was the optical density. The results are presented in percentage of cellular viability with n = 3. As the sterilization method for the coatings, an ethylene oxide stream was used for 4 hours.

### Alkaline phosphatase (ALP) determination

Coated samples in a 24 flat bottom well plate were covered by HOS cells (1.5 × 10^4^ cells/mL) and cultured at 5% CO_2_, 95% humidity at 37 °C. Controls cells were left without coated samples. After 7 and 15 days of incubation, medium was removed from the cells and collected. In addition, cell lysates were obtained by Triton X-100 treatment for 10 min. Removed medium and lysates were centrifuged at 3000 rpm and supernatants were stored at −20 °C until use. The concentration of ALP was determined using a BioSystems kit (ALP-AMP 2-Amino-2-Methyl-1-Propanol Buffer COD 11598). Briefly, supernatants were incubated with a 0.1 M p-nitrophenyl phosphate disodium salt (p-NPP) and plates were reading at 405 nm in a microplate reader Synergy H1 (BioTeK) software Gen5 V3.03 v. The ALP concentration (U/L)(U/L) is calculated using the following formula: $(\Delta {\rm{A}}/\,{\rm{\min }})\times (({\rm{VtX}}{10}^{6})/({\rm{\varepsilon }}\,{\rm{XlX}}\,{\rm{Vs}}))={\rm{U}}/{\rm{L}}(\Delta {\rm{A}}/\,{\rm{\min }})\times (({\rm{VtX}}{10}^{6})/({\rm{\varepsilon }}{\rm{X}}\,{\rm{l}}\,{\rm{X}}\,{\rm{Vs}}))={\rm{U}}/{\rm{L}}$, where $\Delta A/min$ is the average absorbance difference per minute (3 minutes), *ε* the molar absorbance of 4-nitrophenol at 405 nm is 18450, *l* is the lightpath (1 cm), the total reaction volume Vt is 1.02 mL and the sample volume Vs is 0.02 mL.

### Cell adhesion test

HOS cells (1.5 × 10^4^ cells/mL) were placed on the sample coatings and incubated for 96 h as described before. Cells stayed with 2 μg/mL chloroaluminium phthalocyanine for 4 h (for cytoplasm) and 0.5 mg/mL Hoechst 33342 for 10 min (for nucleus). Cells were washed 2 times with neutral PBS and fixed with 2% paraformaldehyde for 10 min. After washing 3 more times, samples coatings were covered with buffered glycerin and examined in an Olympus BX43F fluorescence microscope using the UV2A filter with software CellSens Standard 1.18. All tests were performed in duplicate.

### Statistical analysis

One-way analysis of variance (ANOVA) was applied to determine the differences between the groups used in the cytotoxicity and ALP experiments. Then, Tukey-Kramer test was used to determine statistical differences when comparing two groups. Statistical differences were considered at *p < 0.05 and **p < 0.01.

## Results

### Characterization of MWCNT/CP powders

**Figure 2:**
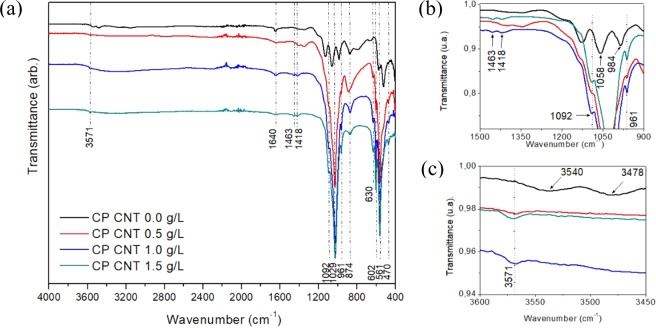
FTIR spectra of (**a**) MWCNT/CP powders with different concentration of multiwalled carbon nanotubes obtained by aqueous precipitation method. Details from parts of the spectrum can be seen in (**b**,**c**).

FTIR spectra of calcium phosphates are shown in Fig. 2. The absorption bands at 3571 cm^−1^ and 630 cm^−1^ originated from O–H stretching and the deformation of P–OH vibration respectively. The band at 1640 cm^−1^ is assigned to H–O–H bending. The bands associated with the ion PO_4_^3−^ are 1092 cm^−1^ (*v*_3_), 1029 cm^−1^ (*v*_3_), 961 cm^−1^ (*v*_1_), 602 cm^−1^ (*v*_4_), 561 cm^−1^ (*v*_4_), corresponding to P–O asymmetric stretching (*v*_3_), P–O symmetric stretching (*v*_1_) and O–P–O asymmetric bending (*v*_4_) respectively. The band for HPO_4_ ion is found at 874 cm^−1^. The band at 470 cm^−1^ is associated with ${\nu }_{2}(P{O}_{4}^{3-})$ out of plane bending. These bands are consistent with a hydroxyapatite-type calcium phosphate (HA)^18–21^, agreeing with CP/CNT 1.0 g/L and CP/CNT 1.5 g/L powders. Likewise, the bands at 1418 cm^−1^ and 1463 cm^−1^ are present for the samples with the highest quantity of nanotubes, due to the vibration of the carbonate group (CO_3_^2−^) that replaces the PO_4_^3−^ groups in the structure of the HA, being an HA carbonated type B, where the carbonate replaces the phosphate groups instead of the hydroxyl groups (type A), because when the carbonated hydroxyapatite is type A, the bands are 1455–1545 cm^−1^^22,23^.

For the CP/CNT 0.0 g/L powder, an IR spectrum consistent with a dicalcium phosphate dihydrate (DCPD-CaHPO_4_.2H_2_O-brushite) was observed. The bands in the different wavelengths have variations with respect to those described above. Two bands pronounced at 3478 cm^−1^ and 3540 cm^−1^ and two more at 3270 cm^−1^ and 3156 cm^−1^ indicated the presence of O–H bonds from the water molecule of brushite. The band at 1640 cm^−1^ is associated with vibration of H–O–H bending, while the bands observed at 1195 cm^−1^, 1126 cm^−1^ and 1058 cm^−1^ are the stretching vibrations of P–O. The absorption bands at 984 cm^−1^, 873 cm^−1^ and 783 cm^−1^ corresponds to the asymmetric stretching vibrations of P–O–P^22,24–26^. A strong band at 520 cm^−1^ and a weaker band at 575 cm^−1^ are due to the binding vibration modes of acid phosphate (H–O–)P=O^22^. A weak band appears at 1346 cm^−1^ corresponding to O–H in plane bending. For the sample CP CNT 0.5 g/L, the bands found in the FTIR are consistent with those described for HA, however, a doublet found at 1346–1407 cm^−1^ indicated the presence of O–H in plane bending for brushite and not to the associated bands for the carbonate ion, suggesting a mixture of HA and brushite for this last sample.

**Figure 3:**
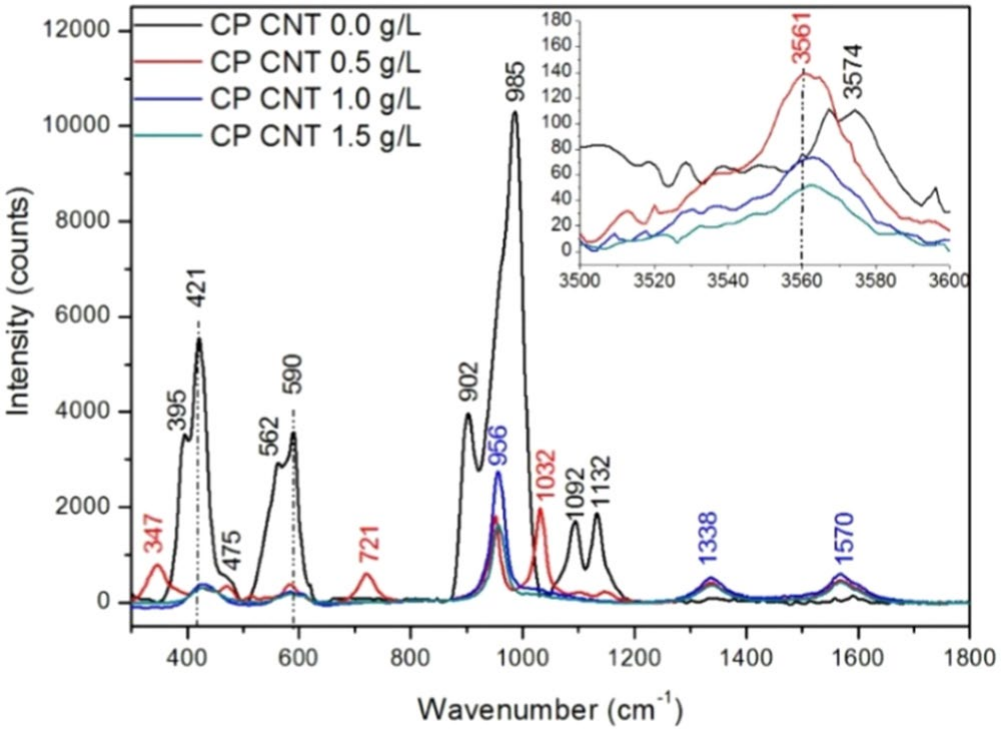
Raman spectra of MWCNT/CP powders with different concentration of multi-walled carbon nanotubes, obtained by aqueous precipitation method. Details from parts of the spectrum can be seen in the inset.

Additional confirmation of FTIR results was gathered by performing Raman spectroscopy on the powder samples (Fig. 3). For brushite, the most intense Raman peak occurred at 985 cm^−1^ which is associated with the symmetric stretch *v*_1_, another band at 590 cm^−1^ was identified for DCPD associated with the vibration of O–P–O(H) bending mode and at 1132 cm^−1^ attributed to HPO_4_^2−^ stretching mode. In addition to the presence of brushite in CP/CNT 0.0 g/L, it was possible to identify in the Raman spectrum bands characteristic of dicalcium phosphate anhydrous (DCPA - CaHPO_4_ - monetite), at wavelengths 395, 421, 475, 562, 902 and 1092 cm^−1^ corresponding to *v*_2_ P–O bending mode (395, 421 and 421 cm^−1^), *v*_4_ P–O bending mode, *v*_3_ stretching mode P–O(H) and *v*_3_ P–O asymmetric stretching mode respectively^24,27,28^. The Raman spectrum for hydroxyapatite has a characteristic peak around 960 cm^−1^, corresponding to the symmetric stretching P–O (*v*_1_), O–P–O doubly degenerate bending mode ~430 cm^−1^, bending mode of the PO_4_ group O–P–O at 585 cm^−1^^22,29^, which agrees with the bands observed in the samples CP/CNT 1.0 g/L and CP/CNT 1.5 g/L. The bands around 3550 cm^−1^ are associated with the vibrations of the hydroxyl group.

For the powder CP CNT 0.5 g/L, Raman modes at 347 and 721 cm^−1^ were in good agreement with the spectrum of Ca(OH)_2_^30^. A band appeared at 475 cm^−1^ was attributed to *v*_2_ P–O bending mode of monetite, at 590 cm^−1^ the vibration band of O–P–O(H) bending mode is registered for brushite with less intensity than CP/CNT 0.0 g/L but higher intensity than CP/CNT 1.0 g/L and CP/CNT 1.5 g/L. Note that the band at 1032 cm^−1^ with a strong intensity was not attributed to brushite, monetite or hydroxyapatite directly. Instead this band can be attributed to the presence of carbonate present in the network, which changes the crystal structure, causing an increase in stoichiometric apatites, which agrees with the results observed via FTIR^31^. The bands at 1338 cm^−1^ (D band) and 1570 cm^−1^ (G band) confirms the presence of carbon nanotubes (–C–C band stretching mode), visible in all samples with CNT. These bands are associated with a disorder-induced mode in the graphite and the purity or symmetry of graphitic structure respectively^32–35^.

Based on the FTIR and Raman spectra, for a calcium phosphate obtained by aqueous precipitation, using fish otoliths as a raw material and without the presence of nanotubes, a brushite-type calcium phosphate is obtained in a mixture with monetite. As the concentration of multiwalled carbon nanotubes changes, the nature of calcium phosphate changes. Thus, for the CP/CNT 0.5 g/L powder, a mixture between brushite, monetite and hydroxyapatite is found with Ca (OH)_2_, finally for CP/CNT 1.0 g/Ly CP/CNT 1.5 g/L carbonated hydroxyapatite.

**Figure 4:**
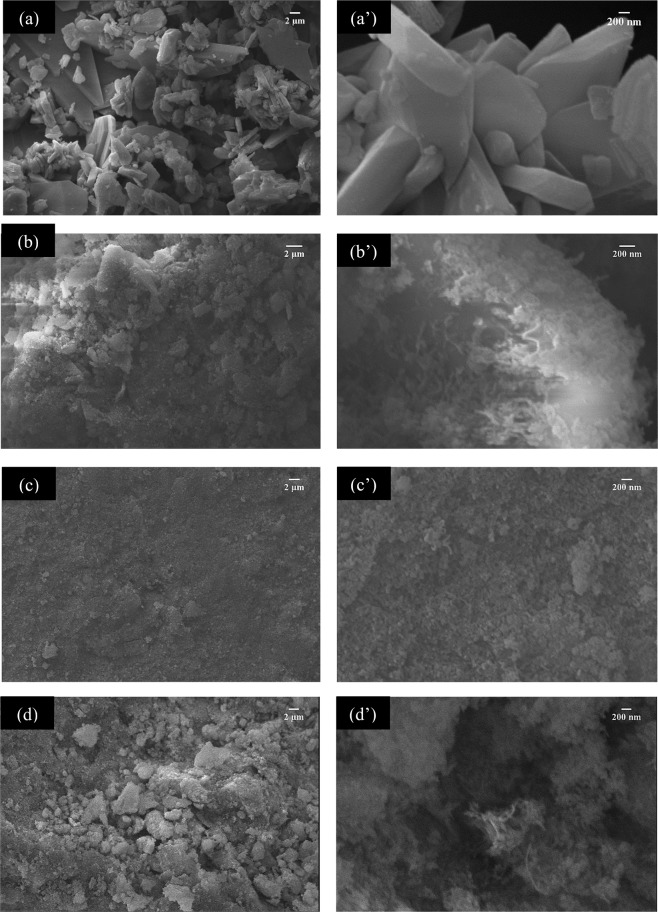
SEM micrographs of calcium phosphate and MWCNT powders **(a**, **a’)** 0.0 g/L CNT, **(b**,**b’**) 0.5 g/L CNT, **(c**,**c’)** 1.0 g/L CNT, and **(d**,**d’)** 1.5 g/L CNT. The original magnification was 2000X for **(a**,**c**,**d)** and 3000X for **(b)**, 20000X for **(a’**,**c’**,**d’)** and 30000X for **(b’)**. Scale bar = 2 µm for **(a**–**d)** and scale bar = 200 nm for **(a’–d’)**. EDS spectrum can be seen in the inset in **(a–d)**.

CP/MWCNT powder was also characterized using SEM as shown in Fig. 4. It was found that the mixture containing carbon nanotubes consist of small particles while the powder composed of brushite and monetite, contains larger particles greater than 200 nm of the self-assembled nanosheets type, which agrees with the morphology of the brushite nucleation^22^. With the addition of carbon nanotubes, the morphology of the ceramic changes towards morphologies of nano-sized rods, which could be verified with TEM. The EDS analysis allowed to find that the Ca/P ratio as 1.31, 1.19, 1.43 and 1.53 for CP/CNT 0.0 g/L, CP/CNT 0.5 g/L, CP/CNT 1.0 g/L, CP/CNT 1.5 g/L respectively, finding the highest values for the powders with the highest amount of CNT. These relationships were calculated using the %At of each element.

**Figure 5:**
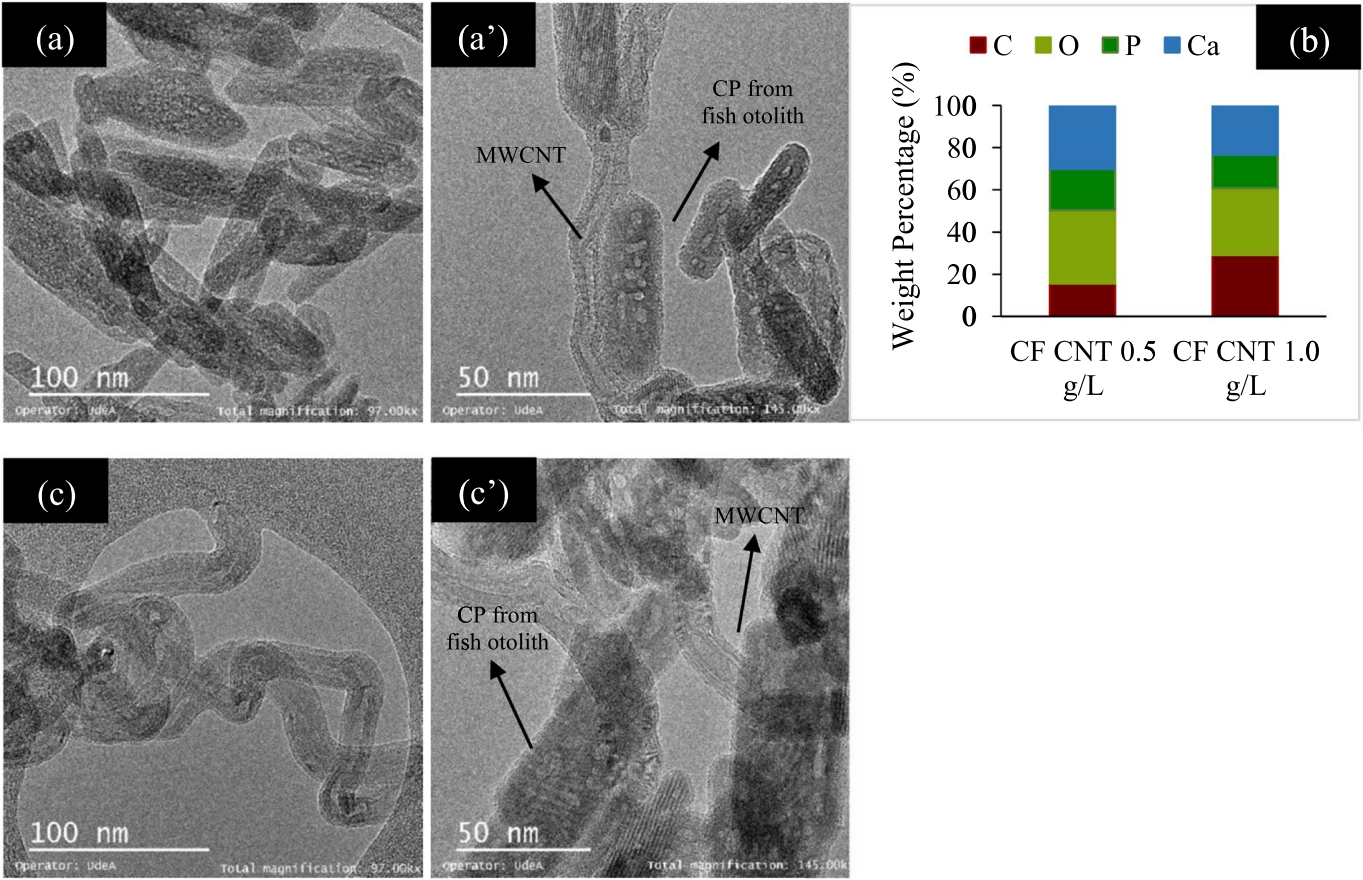
TEM micrographs of calcium phosphate and MWCNT **(a**,**a’)** 0.5 g/L and **(c**,**c’**) 1.0 g/L. The original magnification was 97000X for **(a**,**c)** and 145000X for **(a’**,**c’)**. Scale bar = 100 nm for **(a**,**b)** and scale bar = 50 nm for **(a’**,**b’)**. **(b)** Surface elemental composition (wt %) on respective SEM image using EDS.

According to the TEM analyses (Fig. 5), it is evident that the ceramic structure joins the walls of the nanotubes, even after performing the ultrasound. It is also evident that the diameter of the nanotubes did not change drastically with the acid-base treatment, the walls were not damaged and that the individual ceramic particles are successfully grafted in the multiwalled carbon nanotubes. The elemental surface composition observed in TEM shows an increase in the amount of carbon (% wt) in the CP CNT 1.0 g/L sample compared to a powder sample with a smaller concentration of nanotubes.

### Polycaprolactone/CP/MWCNT coatings

**Figure 6:**
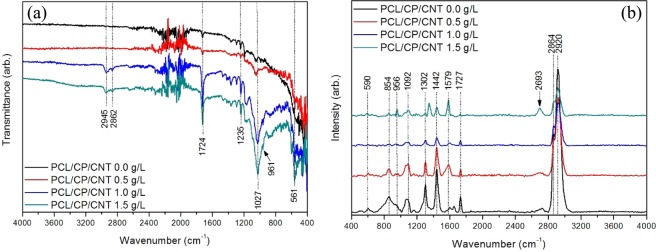
FTIR and Raman spectra of PCL/CP/CNT coatings with different concentration of multi-walled carbon nanotubes prepared by spin coating.

The calcium phosphate/carbon nanotube powder was used to incorporate this reinforcing material into a polycaprolactone polymer matrix. In Fig. 6 the FTIR and Raman spectrum of the composite coating of PCL/CP/CNT made by spin-coating is shown. For the FTIR spectrum (Fig. 6a), the bands at 2945 cm^−1^ and 2862 cm^−1^ are associated with asymmetrical and symmetrical stretching vibrations of the *C*-*H* bonds of methylene groups, the bands at 1724 and 1235 cm^−1^ are associated with stretching *C*=*O* and stretching *C*-*O* of ester groups present in the PCL respectively^4,36,37^. Three other bands at 531, 961 y 1027 cm^−1^ correspond to the vibrations of the group ${{\rm{PO}}}_{4}^{3-}$ in *v*_4_, *v*_1_ and *v*_3_ respectively, as mentioned earlier.

For the Raman spectrum (Fig. 6b), the bands for CH_2_ asymmetric and symmetric stretching are at 2920 cm^−1^ and 2864 cm^−1^ respectively for PCL. The bands at 1727 cm^−1^, 1442 cm^−1^, 1302 cm^−1^, 1092 cm^−1^ and 854 cm^−1^, can be attributed to stretching $({\rm{\nu }}{\rm{C}}={\rm{O}})$, bending $({{\rm{\delta }}{\rm{CH}}}_{2})$, wagging vibration $(\omega C{H}_{2})$, stretching $({\rm{\nu }}{\rm{COC}})$ and stretching $({\rm{\nu }}{\rm{C}}-{\rm{COO}})$ of PCL respectively^38^. These bands described above for PCL have greater intensity for the sample PCL/CP/CNT 0.0 g/L, intensity that decreases as the amount of carbon nanotubes increases. The bands at 956 cm^−1^ and 590 cm^−1^ are related with stretching (*v*_1_*P*-*O*) and the vibration of O–P–O(H) bending mode of the calcium phosphates respectively^24–29^. The band at 956 cm^−1^ is also related with stretching $({\rm{\nu }}{\rm{C}}-{\rm{COO}})$ of PCL. The bands at 1343 cm^−1^ (D band), 1579 cm^−1^ (G band) and 2693 cm^−1^ (G′ band) may be attributed to multi-walled carbon nanotubes, mostly visible in the coating PCL/CP/CNT 1.5 g/L.

**Figure 7:**
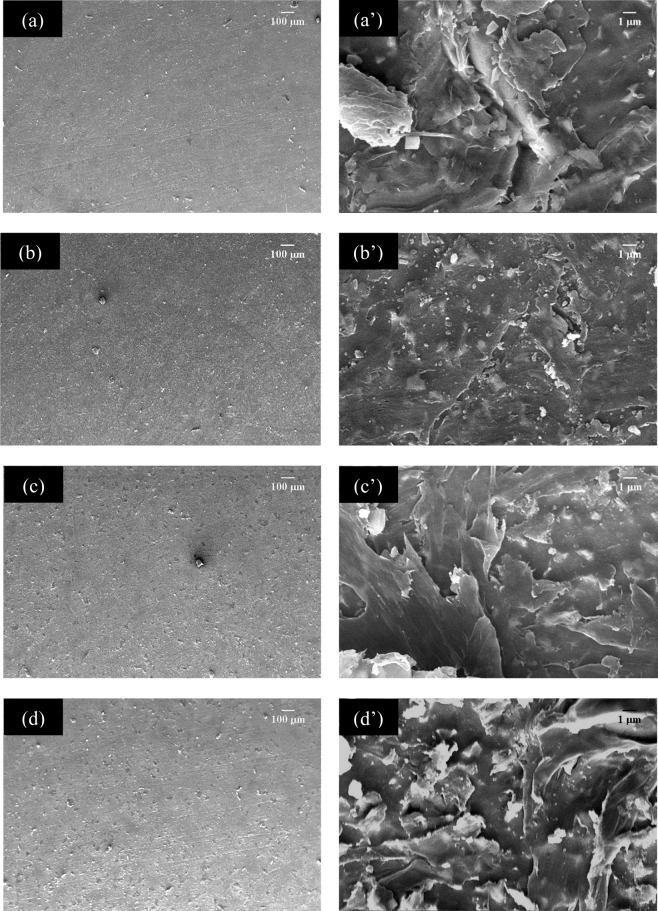
SEM micrographs of polycaprolactone-MWCNT/CP coatings **(a**,**a’)** 0.0 g/L CNT, **(b**,**b’**) 0.5 g/L CNT, **(c**,**c’)** 1.0 g/L CNT and **(d**,**d’)** 1.5 g/L CNT. The original magnification was 50X for **(a**–**d)** and 5000X for **(a’**–**d’**). Scale bar = 100 µm for **(a**–**d**) and scale bar = 1 µm for **(a’**–**d’)**.

Figure 7 are the SEM micrographs showing the distribution of calcium phosphates – MWCNT in the PCL matrix (PCL-CP-CNT). At a magnification of 50×, a more uniform matrix was observed for the sample without nanotubes, however, at a magnification of 5000×, agglomerates were observed in the samples, corresponding to agglomerates of calcium phosphates and carbon nanotubes with calcium phosphates becoming evident in the sample with the highest amount of nanotubes (PCL-CP-CNT 1.5 g/L). It is because of the fact that as the concentration of nanotubes increases, the dispersion of the powder material in the polymer matrix becomes more difficult. Several lines were observed signifying that the material deposition during spin coating did not occur due to the speed used in spin coating, so the coatings were not homogeneous (the optimum spin coating conditions can be found out, but this won’t affect the reported results). However, the biocompatibility of the material depends not only on the chemistry of the material, but also on the surface energy and topography of the material^1^.

### Scratch test of PCL/CP/CNT coatings

**Figure 8:**
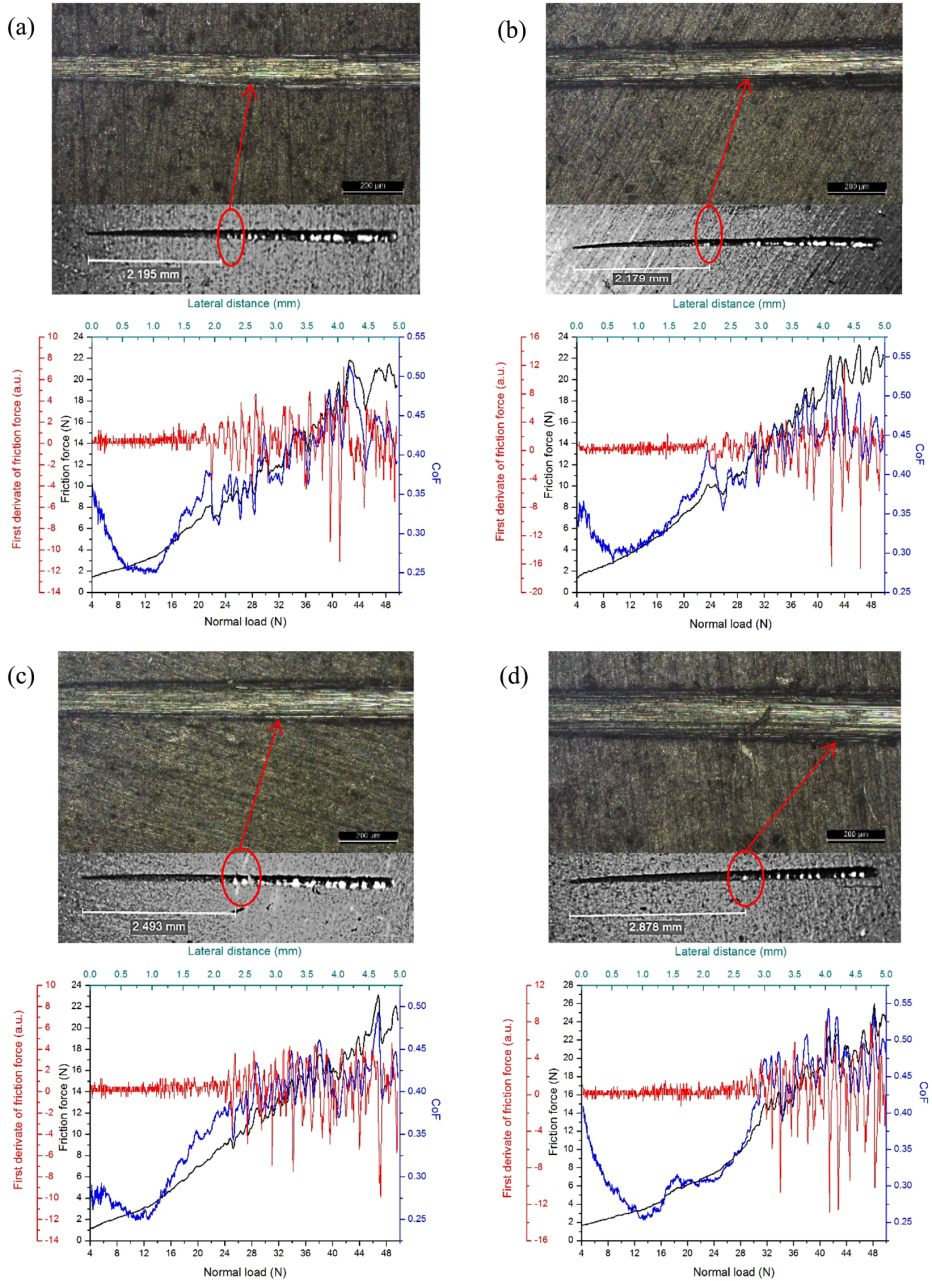
Scratch test of PCL/CP/CNT coatings with different concentration of multi-walled carbon nanotubes. Variation of friction force, first derivate of friction force, friction coefficient versus lateral distance (from 0 to 5 mm) and normal load (from 4 to 50 N). Optical microscope images of scratches, **(a)** PCL/CP/CNT 0.0 g/L, **(b)** PCL/CP/CNT 0.5 g/L, **(c)** PCL/CP/CNT 1.0 g/L and **(d)** PCL/CP/CNT 1.5 g/L. Scale bar = 200 µm for **(a**–**d)**.

Figure 8(a–d) shows the optical microscopy images obtained after the scratch test of different PCL/CP/CNT coatings. The scratch test graphs show the variation of friction force (N), first derivate of friction force, friction coefficient in terms of normal load (N) and lateral distance (mm). The critical load (Lc_3_), calculated with the variation of first derivate of friction force, is the normal load at which the total delamination of coating occurred. A significant variation in the friction force tie in with an abrupt decrease in the coefficient of friction, causes an increase in the variation of the first derivative of the friction force. This normal load point was 21.84 ± 0.50, 23.53 ± 0.29, 25.14 ± 0.51 and 31.72 ± 2.77 N for PCL/CP/CNT 0.0 g/L, PCL/CP/CNT 0.5 g/L, PCL/CP/CNT 1.0 g/L and PCL/CP/CNT 1.5 g/L respectively, and it was taken as the critical load Lc_3_. The increase in normal load is clear as the amount of carbon nanotubes increases. The micro graph images show the near point where the total delamination of the coating exists and worn material at the scratch edge is observed. Furthermore, there is an increase in the coefficient of friction with an increase in the amount of carbon nanotubes, signifying that the PCL matrix is reinforced by the CP and CNT at the point of critical load.

The damage distance was taken from the end of the scratch to the first characteristic of damage observed and converted to a normal load F_z_ where the damage occurs (in this case delamination of the coating) by the following equation^39^.

$${F}_{z}=(\frac{{L}_{s}-x}{{L}_{s}})\ast ({F}_{f}-{F}_{0})+{F}_{0}$$

where L_s_ is scratch length (mm), *x* is the length from the damage feature onset to the end of the scratch (mm) and F_f_ y F_0_ are the final and initial applied normal loads (N) respectively. The results showed a normal load F_z_ of 22.40, 24.24, 25.16 and 31.60 N. Values quite close to those taken as Lc_3_.

**Figure 9:**
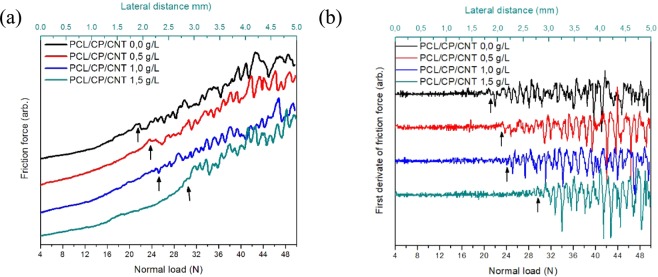
(**a**) Friction force and **(b)** first derivate of friction force versus normal load from 4 to 50 N of PCL/CP/CNT coatings with different concentration of multi-walled carbon nanotubes.

In Fig. 9, one can see the displacement of the critical normal load Lc_3_ (demarcated with arrows) in friction force and first derivate of friction force versus normal load, as a function of an increase in the concentration of CNT revealing that the aggregation of nanotubes to the PCL polymer matrix improves the adhesion of the coating to the Ti6Al4V substrate.

### Toxicity tests

**Figure 10:**
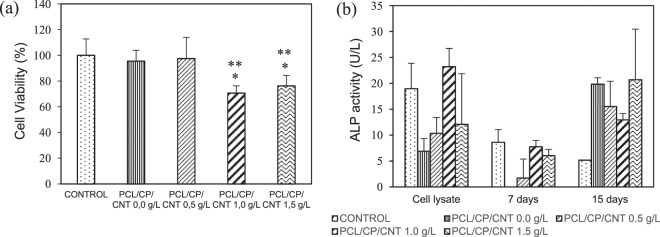
(**a**) Cell viability and **(b)** ALP activity of HOS cells culture in PCL/CP and PCL/CP/MWCNT coatings. Data represents the mean ± standard deviation for n = 3, *p < 0.05 and **p < 0.01 com*p*ared with the controls (statistical test of Tukey-Kramer pair-wise comparison).

The results of cytotoxicity and ALP activity of HOS cells cultured on PCL/CP/CNT coatings are shown in Fig. 10(a). According to ISO 1993–5: 2009 (Biological evaluation of medical devices - Part 5: Tests for *in vitro* cytotoxicity), if the cell viability is reduced to less than 70% of the control, the sample has cytotoxic potential. All the coatings obtained cell viability percentages greater than 70%. An average value of 95.47, 97.43, 70.64, and 76.24% were found for PCL/CP/CNT 0.0 g/L, PCL/CP/CNT 0.5 g/L, PCL/CP/CNT 1.0 g/L and PCL/CP/CNT 1.5 g/L respectively. A considerable decrease was found for the samples with the highest amount of carbon nanotubes (*p* < 0.05 and *p* < 0.01), suggesting that the high concentrations of carbon nanotubes reduced the effect of cell growth. Besides, the MTT test was carried out for 30 days, during this time it is likely that the products dissolved in the supernatants will reduce cell viability. It has been found in other studies that high cell viability values are found in matrices of PCL with low concentration of MWCNT^40^, and that as the exposure time increases, there is a decrease in cell viability with increases in concentration of MWCNT^41^.

### Alkaline phosphatase (ALP) determination

The assay of alkaline phosphatase activity was used as a biochemical marker to determine the differentiation of human osteosarcoma cells both in the cell lysate and in the supernatants on days 7 and 15 Fig. 10(b) shows the values of ALP produced by HOS cells on the polymer matrix coatings of PCL-CP with and without CNT. Low values were found in the supernatants at 7 days, however, when the time increases to 15 days, there is an increase in the production of ALP in greater proportion for the sample with higher concentration of CNT, followed by the sample without CNT. No significant differences in ALP activity in comparison with control cultures were detected (*p* > 0.05).

### Cell adhesion test on the PCL/CP/CNT coatings

**Figure 11:**
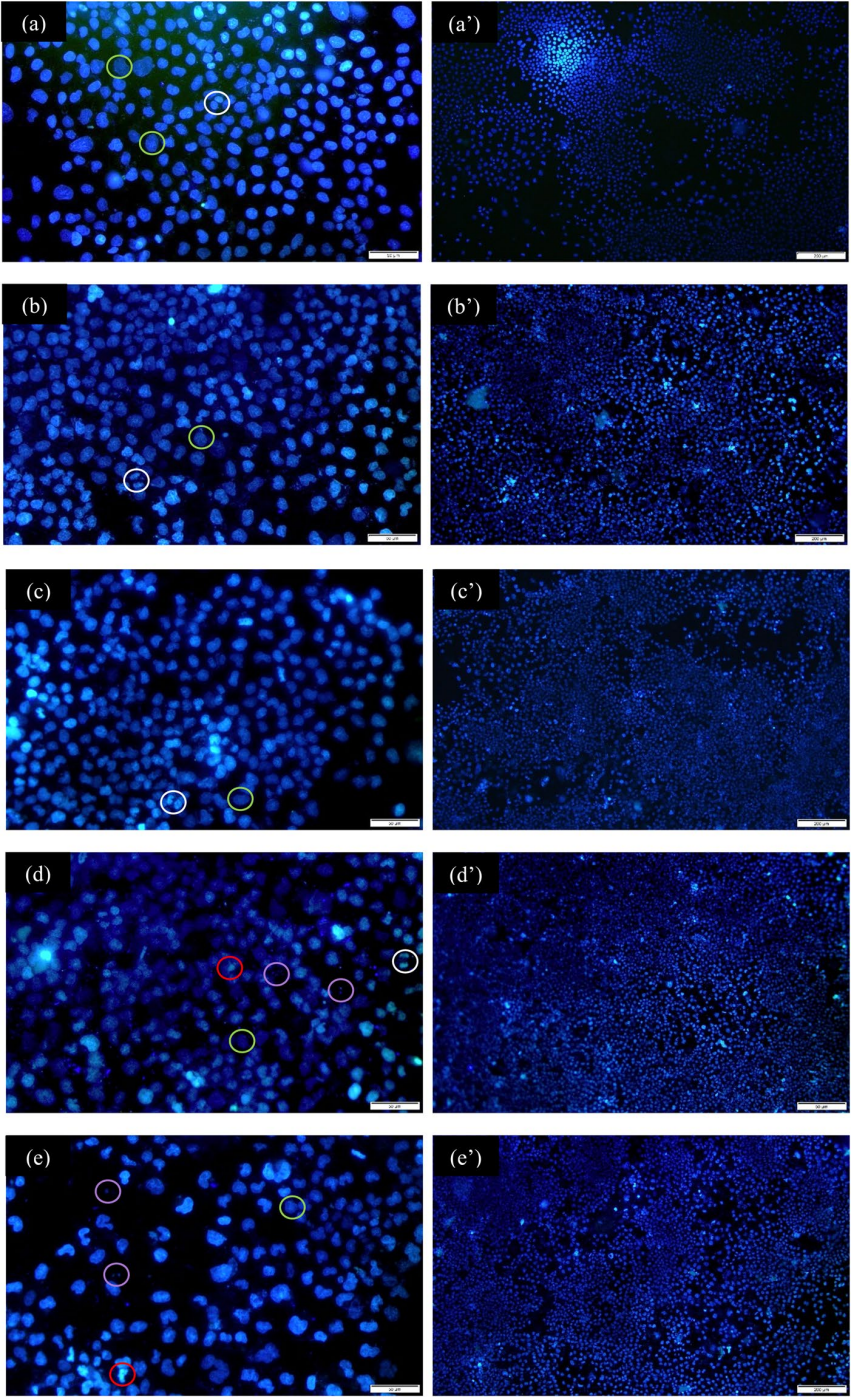
Fluorescence micrographs of polycaprolactone-MWCNT/CP coatings **(a**,**a’**) control, **(b**,**b’**) 0.0 g/L CNT, **(c**,**c’)** 0.5 g/L CNT, **(d**,**d’)** 1.0 g/L CNT and **(e**,**e’)** 1.5 g/L CNT. The original magnification was 40X for **(a**–**e)** and 10X for **(a’**–**e’)**. Scale bar = 50 µm for **(a**–**e)** and scale bar = 200 µm for **(a’**–**e’)**. White circles: mitotic cells, green circles: flattened live cells, red circles: early apoptosis, purple circles: micronucleus.

In Fig. 11, the 10X micrographs shows the area occupied by HOS cells in the control (uncoated) and coated samples. It is evident that is the coated samples contained a larger number of cells compared to the uncoated (control samples), which indicates that the surface modification by coating was favourable to the cellular growth. At 40X magnification, the morphology of the nuclei can be better observed, indicating whether cell growth is normal or abnormal. Cells in early stage of apoptosis were observed in the coatings with the highest concentration of nanotubes, while cells in mitosis were observed in the control, in the coating of 0.0 g/L CNT and 0.5 g/L of CNT. In all the coatings, it was possible to observe flattened live cells and only in the coatings PCL/CP/CNT 1.0 and 1.5 g/L appeared micronuclei, probably formed by an inhibition of the process of mitosis by breaks of the DNA chains^42^, which produces a different nucleus cellular morphology.

**Figure 12:**
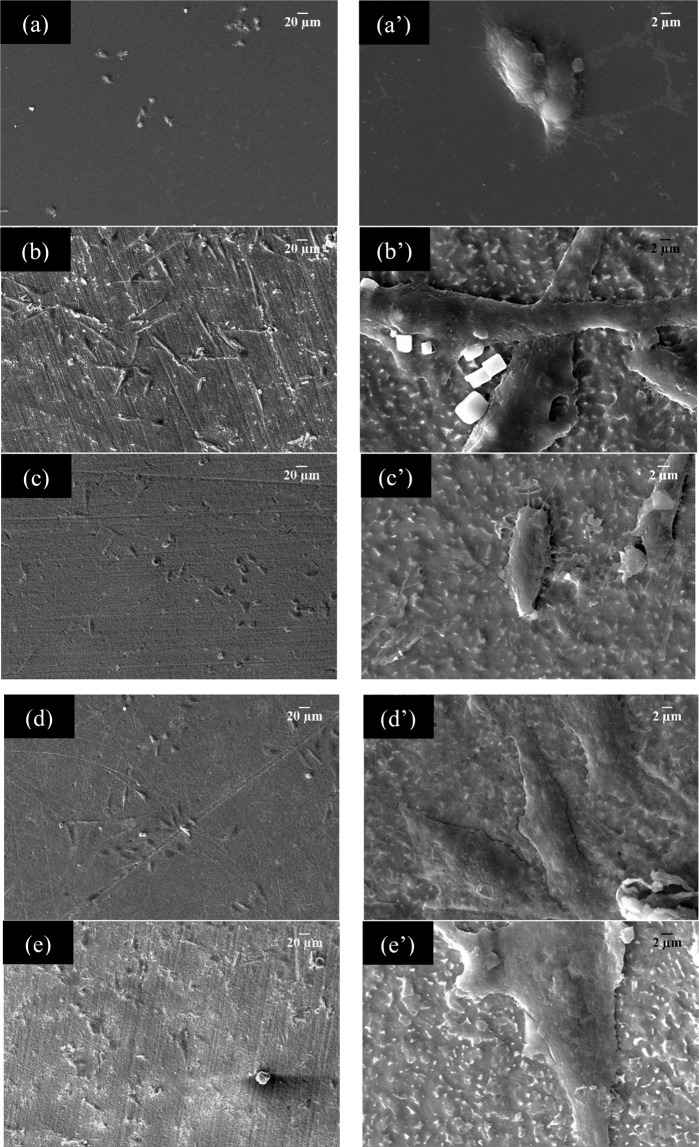
SEM micrographs of polycaprolactone-MWCNT/CP coatings whit HOS cells at 48 h of culture **(a**,**a’)** control, **(b**,**b’)** PCL/CP/CNT 0.0 g/L CNT, **(c**,**c’)** 0.5 g/L CNT, **(d**,**d’**) 1.0 g/L CNT and **(e**,**e’)** 1.5 g/L CNT. The original magnification was 200X for **(a**–**e)** and 2000X for **(a’**–**e’)**.

To clarify these results in the adhesion cell test, SEM tests at 48 hours of cell culture were carried out (Fig. 12). The SEM images show HOS cells adhered to substrates with cytoplasmic extensions some more than others. At 48 hours of culture, the control cells are not as widespread, but it is possible to observe thin cytoplasmic extensions or filopodia^43^ (Fig. 12a,a’). At a magnification of 2000X it is possible to find a different morphology for all surfaces. A very elongated cell is observed for coating with absence of CNT (Fig. 12b,b’). As the concentration of CNT increases, a mostly covered surface is observed and the cells acquire a polygonal morphology (Fig. d,d’,e,e’), showing a differentiation and proliferation different from the control. A relative compatibility of surfaces may be due to the nature of calcium phosphate in the presence of a CNT concentration of 1.0 g/L and 1.5 g/L.

## Discussion

Changes in the structure of calcium phosphate obtained from otolith of Teleost’s fish with carbon nanotubes can be ascribed to the fact that an increase in the amount of carbon nanotubes leads to replacement of PO_4_^3−^ groups by CO_3_^2−^. This results in a phosphate transition of calcium that starts in a brushite-monetite mixture, passing through brushite-monetite-hydroxyapatite and Ca(OH)_2_ by decreasing the 1092 cm^−1^ and 1132 cm^−1^ Raman bands and increasing the 1032 cm^−1^ and 956 cm^−1^ bands. It was found that the samples with the highest amount of nanotubes contained carbonated hydroxyapatite, where the Raman band at 3561 cm^−1^ was diminished by the presence of hydroxyl groups and not by the presence of water in the brushite, in addition to the complete elimination of the band 1032 cm^−1^, these transitions are favorable, since, in bone apatite contains 2 to 6% by weight of CO_3_^2−^ which substitutes PO_4_^3,44^. These substituted hydroxyapatite type B powders are more amorphous exhibiting nanometric particle size and have better bioactivity^22^.

Although carbonated hydroxyapatite was obtained in the samples with the greatest concentration of nanotubes, on the other hand, it is true that the presence of CNT modifies the cellular response, towards less-desired results. The results indicate that the surfaces PCL/CP/CNT 0.0 g/L and PCL/CP/CNT 0.5 g/L could behave better in an animal model for biological tests, however, what is obtained in these samples is brushite and monetite and not carbonated hydroxyapatite. Brushite is a biomaterial widely used in osteogenesis, has been detected in fracture callus, in calcified forms, does not induce inflammation and has excellent biocompatibility, its resorption rate is lower than HA, it also has the ability to increase ion levels of calcium and phosphate near the implant-tissue interface and can become a less soluble calcium phosphate such as HA^22,45^. The process of dissolution of brushite and conversion to hydroxyapatite is shown in Eq. 1^46^. The dehydration of brushite can easily convert it into monetite (Eq. 2). On the other hand, monetite has demonstrated its capacity for bone formation in bone defects^47^, it has a resorption rate like that of brushite, *in vitro* and *in vivo* tests it can be converted to octacalcium phosphate^48^.

1 $$10CaHP{O}_{4}.2{H}_{2}O\to C{a}_{10}{(P{O}_{4})}_{6}{(OH)}_{2}+4{H}_{3}P{O}_{4}+18{H}_{2}O$$

2 $$CaHP{O}_{4}.2{H}_{2}O\to CaHP{O}_{4}+{H}_{2}O$$

With respect to biocompatibility tests, it is known that the toxicity of nanotubes depends on several factors such as surface load, diameter, length, agglomeration, number of layers, concentration and dose. On the other hand, the addition of calcium compounds can also modify cell viability. In a study by Duarte *et al*., Obtained scaffolds of PCL, β-TCP and dexamethasone using CO_2_ as a foaming agent, obtaining supernatants for only 24 hours for MTT testing. They found that there is a decrease in cell viability from ~130% for PCL to ~122% for PCL/β-TCP and ~99% for PCL/β-TCP/5% dexamethasone, concluding that, although cell viability changes, surfaces do not affect the metabolic activity of L929 osteoblast cells^49^.

An increase in the production of ALP, associated with lower viability values, where a low proliferation rate is probably due to the fact that the cells begin a stage of bone differentiation^50^. This result is consistent with those expressed by Lee *et al*., where show that the expression of ALP in polymeric matrices of PCL with and without black phosphorus (BP, white phosphorus allotrope when treated at high pressures), does not have significant differences evaluated at days 1, 7, 14 and 21, and that the presence of BP slightly increases the ALP expression of MC3T3-E1 osteoblast cells^51^. In all coatings, it was possible to observe extended cell nuclei on the surface, without condensed and fragmented nuclei, which are typical morphological characteristics of an apoptotic cell^52–54^. This is related to the coatings that presented the lowest cell viability (PCL/CP/CNT 1.0 and 1.5 g/L), since as mentioned earlier, in this type of PCL coatings, cell viability decreases with increases in the CNT concentration.

According to the cell adhesion test, an increase in the concentration of nanotubes modifies the morphology of the cell nucleus and the viability, however, none of the proposed coatings exceeds the threshold to be considered potentially cytotoxic. An increase in ALP values were found for PCL/CP/CNT 1.5 g/L > 0.0 g/L > 0.5 g/L coatings in supernatants after 15 days of cell culture, these values are not related to the number of cells, these high values specifically of CNT 1.5 g/L may be due to altered cell proliferation and differentiation^55^. At 48 hours of cell culture, SEM micrographs with cells show different morphologies of HOS cells due to the nature of CNT functionalized with CP. The change in the chemistry of the CP with CNT substantially changes the behavior of cells, where the more polygon type morphology is presented for coatings with a higher concentration of CNT.

The addition of calcium phosphate improved the adhesion of the coating with the material. The increase in CNT greatly improves the adhesion of the coating to the substrate. A minimum critical load of 21.84 ± 0.50 N indicates that the presence of phosphates also increases the resistance of the polymer coating, like the results found by Catauro *et al*.^56^. The results indicate that by increasing the adhesion to the substrate by incorporating CP and CNT, a minor release of components to the medium occurs, which makes the HOS cells have a good behavior in terms of cell viability and activity by measurement of ALP. At a certain point of concentration of carbon nanotubes, there is a decrease in cell viability and changes in nucleus morphology. According to other studies, the differentiation and mineralization of osteoblastic cells is inhibited by the presence of carbon nanotubes^43^, since depending on the properties of the nanotubes, these can be deposited in the bone for long periods of time or be internalized by the cells located in the lysosomes and the cytoplasm or accumulate in the liver for a period of three months with low toxicity. for example, long (needle-shaped) CNT, or greater than 800 nm in length, are related to toxicity since they induce a greater degree of inflammation (increased pro-inflammatory cytokines such as IL-1), granuloma formation and damage to DNA level than the shortest CNT^57–59^.

As for the calcium phosphate present in the composite, it has been found that partial dissolution of HA increases the local concentration of phosphate ions (HPO_4_^2−^, PO_4_^3−^) and calcium (Ca^2 +^), followed by carbonate phosphate precipitation of calcium, these precipitates induce bone growth towards the implant by attracting osteoblasts, followed osteoclasts dissolve the carbonaceous precipitates in the native bone, in the defects of low resorption of HA^60–62^. An *in vivo* model is necessary to explain the mechanism of degradation of each component in a biological system, with this research and *in vitro* tests, it is concluded that there is compatibility of HOS cells in this type of composite coatings.

It is concluded that not only the chemistry of the material affects the biocompatibility, but the morphology, roughness, physical agents such as the surface charge, adherence of the coating to the substrate and phase conversion in the solution determine the behavior of the material *in vitro*.

## Conclusions

This work reports the development and testing aspects of a novel biocomposite coating synthesised by mixing PCL polymer with layers of calcium phosphate (hydroxyapatite, brushite and monetite) synthesized from a biomineral called otolith extracted from Teleost fish (Plagioscion Squamosissimus) and functionalised multiwalled carbon nanotubes in different concentrations (0.5, 1.0 and 1.5 g/L). The biocomposite coating was deposited on an osteosynthesis material Ti6Al4V by spin coating and functional tests were performed. Multiwalled carbon nanotubes used in concentrations of 0.5 g/L, 1.0 g/L and 1.5 g/L were functionalized with nano-sized calcium phosphates prepared by aqueous precipitation from Teleost’s fish otolith. An increase in the concentration of carbon nanotubes shows a change in the chemistry of calcium phosphates, thus, it establishing that the brushite-monetite and brushite-monetite-hydroxyapatite phases coexist for the powders PCL/CP/CNT 0.0 g/L and PCL/CP/CNT 0.5 g/L respectively, while for PCL/CP/CNT 1.0 g/L and PCL/CP/CNT 1.5 g/L the carbonated hydroxyapatite phase was found. FTIR, Raman, TEM and SEM results collectively demonstrates that the resultant powders of CP/CNT were well cross-linked to the PCL polymer matrix. From the scratch tests, it was observed that an increase in the MWCNT produces an increase in the Lc_3_ normal load at which the delamination of the coating exists, showing a strong indication of an improved adhesion to the substrate. The compatibility of the coating changes with the use of CP/CNT powder material containing high concentrations of CNT, decreasing cell viability and modifying cell morphology, finding early apoptosis in HOS cells for PCL/CP/CNT 1.0 g/L and PCL/CP/CNT 1.5 g/L compared with the control. *In vitro* assays of bioactivity by alkaline phosphatase with HOS cells show that ALP values benefit from longer evaluation times with higher values for PCL/CP/CNT coatings 0.0 g/L and PCL/CP/CNT 1.5 g/L. It was concluded from these overall results that the newly developed biocoating composed of polycaprolactone, calcium phosphates from otolith and carbon nanotubes in low concentrations, is a potentially future material for use in regeneration and treatment of bone diseases.

## Data Availability

All data generated or analysed during this study are included in this published article and its Supplementary Information Files.
